# Cerebral venous thrombosis: a Moroccan retrospective study of 30 cases

**DOI:** 10.11604/pamj.2014.17.281.165

**Published:** 2014-04-14

**Authors:** Zouhayr Souirti, Ouafae Messouak, Faouzi Belahsen

**Affiliations:** 1 Neurology Department, University Hospital of Fez, Morocco

**Keywords:** Cerebral venous thrombosis, Heparin, Angio-MR

## Abstract

Cerebral venous thrombosis (CVT) is a rare origin of stroke, the clinical presentation and etiologies vary. The prognosis is shown to be better than arterial thrombosis. Magnetic Resonance Imaging (MRI) and MR Venograpgy (MRV) are currently important tools for the diagnosis. We studied 30 cases of CVT diagnosed in the department of neurology at the University Hospital of Fez (Morocco). Patients diagnosed with CVT signs between January 2003 and October 2007 were included in the study. Cerebral CT-scan was performed in 27 cases (90%) while the MRI examination was done in 18 patients (67%); and most patients (90%) received anticoagulant therapy. The mean age of our patients was of 29 years (age range between 18 days and 65 years). A female predominance was observed (70%). The clinical presentation of patients was dominated by: headache in 24 cases (80%), motor and sensory disability in 15 cases (50%), seizures in 10 cases (33%), consciousness disorder in 10 cases (33%). CVT was associated to post-partum in 10 cases (33%), infectious origin in 8 cases (26%), Behçet disease in 2 cases (7%), pulmonary carcinoma in 1 case, thrombocytemia in 1 case and idiopathic in 7 cases (23%). The evolution was good in 20 cases (67%), minor squelaes were observed in 6 patients (20%), while major squelaes were observed in 2 cases. Two cases of death were registered. The CVT is a pathology of good prognosis once the diagnosis is promptly established and early heparin treatment initiated.

## Introduction

CVT is a rare origin of stroke. This pathology was considered an infectious disease for a long period. In addition, it was assumed to be affecting the superior sagittal sinus dragging to death. Anticoagulants were contraindicated while the diagnosis was established at the autopsy stage. Currently CVT is a disease with various clinical presentations and etiologies. MRI and MRV are the key diagnosis tools. Heparin is the first-line treatment. The prognosis is better compared to arterial thrombosis with mortality well below 10%. The goal of this study is to describe and discuss different features of this pathology including frequency, clinical model, diagnosis and outcome.

## Methods

**Figure 1:**
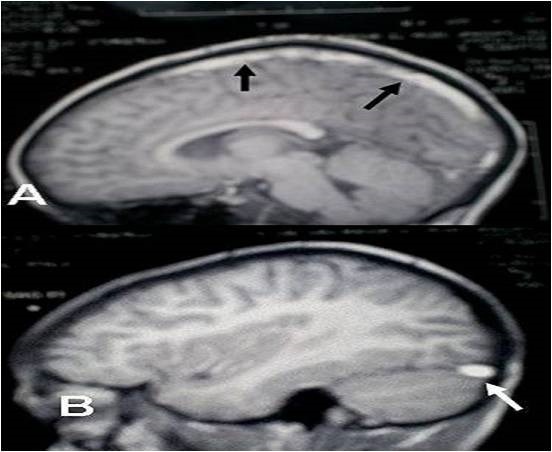
(case 21) Sagittal MR T1 sequences without Gadolinium injection showing: hypersignal of thrombosed SSS on the right (black arrows) (A), hypersignal of thrombosed LLS on the left (white arrow) (B)

We studied 30 cases colligated in the department of neurology of the University Hospital of Fez (Morocco). Patients were recruited from January 2003 to October 2007 (58 months). All patients demonstrating clear radiological signs of CVT were included. The cerebral CT-scan was performed in 27 patients (90%). The period between onset and CT scan varied from 1 day to 45 days. The MRI examination was achieved in 17 patients (56%). Ninety percent of patients (90%) received anticoagulant therapy. Considering the CT scan without injection of contrast agents, the inclusion criteria was spontaneous hyperdensity or cord sign; while contrast agents was injected the empty delta sign and empty lateral sinus sign were the inclusion criteria. On MRI, inclusion criteria were hypersignal on T1 and T2 sinuses (Figure 1), abnormal defect or rupture of the sinus in MRV except two cases of deep cerebral venous thrombosis who presented indirect signs of thrombosis of deep veins. The MRI T1 and T2 weighted were done in axial, sagittal and coronal slices. Axial FLAIR and echoplanar susceptibility-weighted (T2*), and T1 with gadolinium injection were also achieved. Our patients have had the MRI and MRV between 5^th^ and 30^th^ day of onset. The diagnosis of sinus thrombosis was based on hyperintense signal on T1 and isointense or hyperintense signal on T2, and by the absence of flow on MRV. The diagnosis using the CT scan was based on the presence of: empty delta sign or empty lateral sinus sign.

The CT scan was sufficient for establishing the diagnosis in 13 cases (43%). Hence, the MRI examination was not performed for these patients. The MRI examination was achieved directly and without earlier CT-scan in 3 patients (10%). Fourteen patients (47%) have benefited of both CT scan and MRI.

## Results

The mean age of our patients was 29 years old, ranging from 18 days to 65 years old. High predominance of female involvement was noticed with sex ratio of 2.3. The average period between the onset of symptoms and consultation was 18 days. The Table 1 summarizes patients’ data.

**Table 1:** Patients data

N	Age, gender	Clinical symptoms	Causes and risk factors	Topography	Parenchyma involvement	Outcome
1	11, M	ICH, P, IIIrd np, VIth np, exophtalmos,	Severe staphylococcal of the face	SC	no	normal
2	27, F	ICH, P, motor deficit, DC	Post-partum, otomastoiditis	SSS, SLD	yes	Motor deficit
3	60, M	Seizures, motor deficit, DC, fever	absent	SLD	yes	normal
4	24, F	Ptosis, headache, IIIrd np, VIth np, exophtalmos, DC, fever	Severe staphylococcal of the face	SC	no	IIIrd np, VIth np
5	17, F	ICH, P, VIth np, exophtalmos, fever,	Probable local infection	SC	no	death
6	27, F	ICH, P, seizures, motor deficit,	Post-partum	SSS	yes	seizures
7	43, M	Seizures, motor deficit,	Absent	SSS, SLG	yes	normal
8	28, F	ICH, seizures, motor deficit, DC	Post-partum	SSS	yes	death
9	21, F	ICH, fever	Post-partum	SSS	No	normal
10	17, M	ICH, P,	Behçet disease	SLD	No	normal
11	18, F	Headache, status epilepticus, motor deficit, DC, fever	Post-partum	SSS		
SLD	Yes	normal				
12	18, F	ICH, P, fever	Otomastoiditis	SLG	No	normal
13	46, M	ICH, IIIrd np, VIth np	Severe staphylococcal of the face	SC	No	Blindness, IIIrd np, VIth np
14	54, M	ICH, P, motor deficit	Pulmonary carcinoma with metastasis	SLD	yes	death
15	25, F	ICH, P, status epilepticus, DC, fever	Post-partum	SSS, SLD	yes	normal
16	24, M	Headache, fever, IIIrd np, VIth np exophtalmos	Ethmoiditis	SC	no	normal
17	65, F	DC	Miliary tuberculosis	SLD	no	normal
18	25, F	Status epilepticus, DC, fever	absent	Basilar veins, ICV	yes	Cognitive dysfunction, motor deficit
19	13,M	ICH, P	otomastoiditis	SLD	No	normal
20	40, F	Headache, motor deficit	Post-partum, epidural analgésia	Cortical vein	No	normal
21	7, F	ICH, P, fever	Absent	SSS, SLD, SLG	No	normal
22	55	ICH, motor deficit, DC	Absent	SLG	Yes	death
23	18 months, F	Status epileptcus, hyporeactivity	Protein C deficiency	SSS	yes	Pyramidal hypertonia
24	40, F	Headache, seizures, motor deficit	Post-partum	SSS	yes	Motor deficit
25	19, F	Headache, DC	Post-partum,	SLD	No	normal
26	18, F	ICH, P	Essential thrombocytemia	SSS	No	normal
27	44,F	ICH, motor deficit, fever	absent	SSS, RLS	no	normal
28	24,F	Headache, DC	Post-partum	SSS, LLS	yes	normal
29	28,M	Motor deficit	Behcet disease	Basilar veins	yes	Motor deficit
30	23,F	ICH, seizures, DC, fever	absent	SSS	yes	normal

IIIrd np, third nerve paralysis; DC, disorder of consciousness; ICH, intracranial hypertension; ICV, internal cerebral veins; P, papilledema SSS, superior sagittal sinus; LLS, left lateral sinus; RLS, right lateral sinus; CS, cavernous sinus; F: feminine; M: masculine.

### Clinical presentation

**Figure 2:**
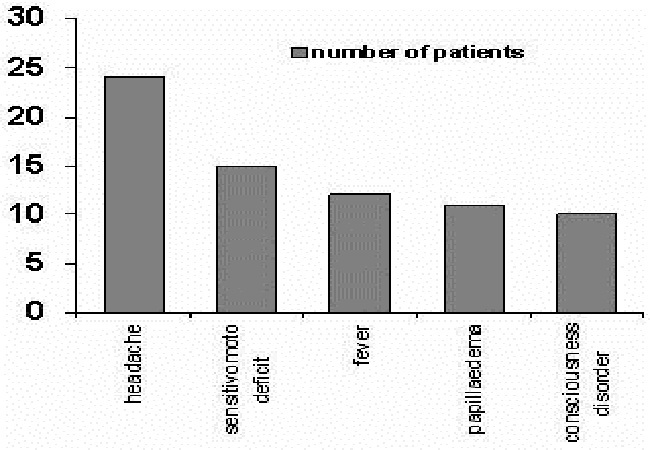
Principal clinical signs of the 30 patients

The mode of onset of symptoms was subacute in 50% of cases, acute in 47% and progressive in one case (3%). The clinical presentation was variable, but the most common symptoms were headaches in 24 cases (80%), motor and sensitive disability in 15 cases (50%), seizures in 10 cases (33%) including two cases of status epilepticus, consciousness disorder was noticed in 10 cases (33%) and fever (40%) (Figure 2).

### Biological and Radiological Examinations

**Figure 3:**
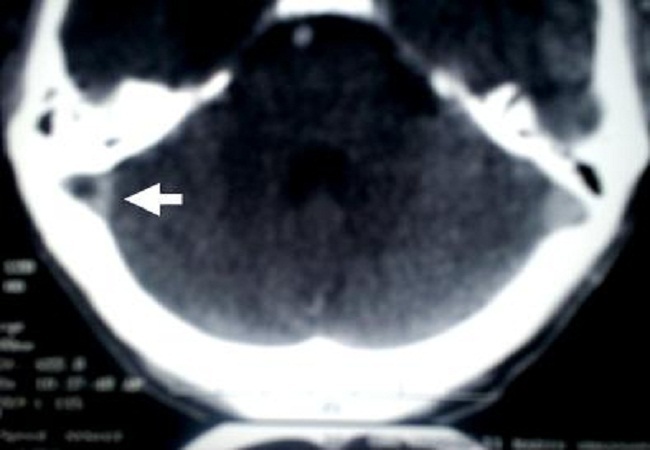
(case 14) CT scan after contrast product injection, axial sequence of the posterior fossa showing empty right lateral sinus (arrow)

**Figure 4:**
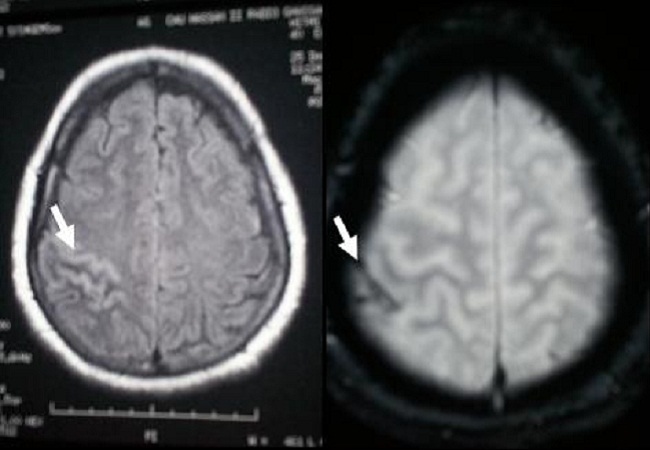
(case 20): FLAIR MR cerebral axial sequences (on the left) showing a hypersignal at the level of the ascending frontal gyrus (arrow) and in T2* (on the right) showing a hyposignal of a cortical vein (arrow) which is thrombosed

The biological examination showed anemia in 13 cases (43%), hyperleucocytosis in 8 cases (27%), raised erythrocyte sedimentation rate in 11 cases (37%), thrombophilia was recorded in four patients including low S-protein in three cases and C-protein in one case. The CT-scan showed direct signs such as empty lateral sinus in 11 cases (37%) (Figure 3), cord sign in six cases (20%), empty delta sign in seven cases (23%). The indirect signs were cortical hypodensity in 10 cases (33%), subcortical hypodensity in 12 cases (40%), parenchymal hematoma (1 case), enhancement of wall sinuses after contrast agent injection in 9 cases (30%), multiple and grouped hyperdensities in six cases (20%). 14 patients benefited both CT scan and MRI examinations. The CT scan performed diagnosis in five cases (35%), confirmed by MRI associated with MRV. The CT scan was normal in three cases (21%). The MRI associated with MRV was more efficient in demonstrating the lesion compared to CT in 9 patients (65%) (Table 2). The CT-scan and/or MRI including the MRV showed the CVT in the sagittal superior sinus (SSS) in 15 cases (50%), right lateral sinus (RLS) in 12 cases (40%), left lateral sinus (LLS) in 3 cases (10%), cavernous sinus (CS) involvement in 5 cases (16%), deep cerebral vein in two cases (7%), and cortical cerebral vein in one case. The diagnosis of cortical cerebral vein thrombosis was also demonstrated a hyposignal on echo-planar susceptibility weighted (T_2_*) images (Figure 4). The diagnosis of deep cerebral vein thrombosis was performed by indirect signs on MRI, and theses cases have shown normal deep venous system on MRV images.

**Table 2:** The 14 patients with both CT scan, MRI and MRV

Case	CT scan	MRI	MRV	Thrombosed sinus or vein
1	Absence of bilateral CS enhancement,	Absence of bilateral CS enhancement, aneurysm of left intracavernous carotid	normal	CS
3	Cortical and subcortical hypodensity	HS T1,T2 RLS; venous infarction	Amputation RLS	RLS
5	normal	Absence of bilateral CS enhancement, venous infarction.	Amputation LLS (hypoplasia).	CS
6	Cortical and subcortical hypodensity, multiple grouped hyperdensities	IsoT1, hypoT2 SSS; hemorrhagic infarction	Amputation SSS	SSS
14	Empty lateral sinus sign, enhancement of sinus wall, cortical and subcortical hypodensity	HS T1,T2 RLS; multiple metastasis	Amputation RLS	RLS
16	Absence of bilateral CS enhancement, convexity and enhancement of CS wall	Absence of right CS enhancement; aneurysm of right intracavernous carotid	Normal	CS
17	Cord sign, empty right lateral sinus sign.	HS T1,T2 RLS; multiple tuberculoma.	Amputation RLS.	RLS
18	bilateral venous infarction (Rosenthal vein and intern cerebral vein).	Partiel obstruction of LLS (T1 Gadolinium), bilateral venous infarction (Rosenthal vein and intern cerebral vein).	Normal	Rosenthal vein and intern cerebral vein
20	normal	HS FLAIR, hyposignal T2* of a cortical vein.		
	normal	Cortical vein		
21	Empty delta sign, empty lateral sinus sign (right and left)	HS T1,T2 RLS, LLS, SSS	Amputation SSS, RLS, LLS	SSS, RLS, LLS
24	Hematoma	Hematoma	Defects SSS	SSS
25	Empty right lateral sinus sign	HS T1, T2 RLS	normal	SSS
26	normal	HS T1, isosignal T2 SSS		
28	Cortical and subcortical hypodensity, multiple grouped hyperdensities	hemorrhagic infarction	Defects SSS, LLS	SSS, LLS

HS, hypersignal intensity; SSS, superior sagittal sinus; LLS, left lateral sinus; RLS, right lateral sinus; CS, cavernous sinus

### Etiology

**Figure 5:**
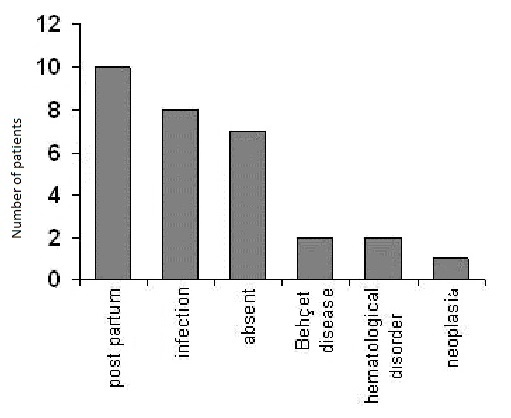
Etiologies of the 30 patients

All patients underwent systematically an investigation of the thromboembolic antecedent, clinical examination, blood cells enumeration, prothrombin time (PT), activated partial thromboplastin time (APTT), erythrocyte sedimentation rate (ESR) and a thoracic x-ray. The post-partum was the most frequent cause of CVT in our series consisting of 10 cases (33%); infectious origin was seen in 8 cases (26%), four cases of otomastoiditis, three cases of severe staphylococcal of the face and these patient have had delayed treatment, one case of ethmoiditis, one case of miliary tuberculosis; Behcet disease in 2 cases (7%); pulmonary carcinoma in 1 case; essential thrombocytemia in 1 case and thrombophilia in one case (deficiency in protein C) (Figure 5). Finally, in 7 cases (23%), the etiology of the CVT could not be established.

### The treatment

The treatment of intracranial hypertension was based on carbonic anhydrase inhibitor or mannitol (63%), and the lumbar puncture was performed to remove the cerebrospinal fluid (23%). Most of our patients (90%) received heparinotherapy (low-molecular-weight heparin 68% or standard heparin 32%) followed by oral anticoagulant for 6 months. Four patients (10%) did not receive heparin, two have had a thrombosis of the cavernous sinus complicated by a carotid aneurysm; hence the anticoagulation was contraindicated. The third patient was a newborn of 18 days.

Antibiotherapy was administrated to patients with infectious origin (47%). Antiepileptics (27%) were prescribed for cases of seizures. We used steroids (prednisone 1mg/kg/day) in two cases of Behcet disease. Allopurinol (300mg/day) and hydroxycarbamid (1000mg/day) were used in cases of essential thrombocytemia. Two patients with severe disorder of consciousness were hospitalized in reanimation department; one of them has died in this department. The surgery was not the treatment of choice in any of the reported cases.

### The outcome

After treatment, a full recovery was noticed in 18 cases (60%). Sequelae were observed in 8 patients (27%), these consisted of blindness in one case associated to cavernous sinus thrombosis complicated by severe staphylococcal of the face. Hemiparesis was noticed in 3 cases, tetraplegia associated to cognitive dysfunction was recorded in case of deep cerebral veins thrombosis. Epilepsy was noticed in a case stabilized using carbamazepin; ophtalmoplegia after a cavernous sinus thrombosis; pyramidal hypertonia in the newborn of 18 days. Three patients died (10%) including one patient in the post partum, one of cavernous sinus thrombosis and one idiopathic.

## Discussion

Cerebral venous thrombosis is a rare type of stroke that might occur at any age. The diagnosis of CVT requires a visualization of the thrombosis, which is commonly demonstrated by cerebral MRI and MRV. The CT-scan might be helpful but does not allow establishing the diagnosis.

CVT affects about five people per million per year and represents 0.5% of all stroke [1]. CVT might occur in any stage of life. Most authors agree on the predominance of CVT in young subjects, this was also revealed in our study. Similar agreement was found for the gender ratio [1]. In our series, we found a sex ration of 2,3. The female predominance is explained by the high frequency of CVT in the post-partum. The clinical presentation is determined by the patient age, the delay between the onset and the hospitalization, the location of the thrombosis and the occurrence of cerebral parenchymal lesions.

Headaches represent the main and first symptom of CVT retrieved in most studies. CVT with isolated headache is possible, without intracranial hypertension, subarachnoid haemorrhage, or meningitis signs and symptoms[2, 3]. Other symptoms are partial and generalized seizures, motor or sensitive deficits, altered consciousness, and papilloedema which might be associated to other symptoms [4]. Motor or sensitive deficit, altered consciousness and seizures are frequent in our study and agrees with earlier study (Einhäupl 1990), this was mostely related to delayed patients consultation. The 18 days newborn patient has shown a generalized seizures and hyporeactivity as main symptoms of the SSS thrombosis. The case number 20 has shown isolated cortical cerebral vein thrombosis revealed by motor deficit without seizures association.

In SSS thrombosis, signs are dominated by the unilateral or bilateral motor deficit seizures and consciousness disorder. On the other hand, the isolated intracranial hypertension syndrome is not frequent in SSS thrombosis. However, CVT of the LS is often revealed by an isolated intracranial hypertension syndrome. When the left LS is affected, an aphasia occurs, thereafter, a patient can present with Wernicke aphasia and partial seizures.

The clinical manifestations of deep CVT are more severe, it often presents with coma and bilateral motor deficit. These manifestations have been noticed in case number 18, while the case number 29 presented only a unilateral motor deficit. Seizures are more frequent during the CVT comparing to the other types of stroke. Seizures occur mainly in cases of parenchymal lesions, SSS thrombosis and in patients having motor or sensitive deficit [5].

Most of the routine blood assessment does not have diagnostic value for CVT. However, they may help establish its etiology. Several studies confirmed the elevation of the D-dimers during the CVT but their predictive negative value seems only interesting for patients having signs of encephalic reach. Therefore, an absence of D-dimers elevation in case of isolated headache can not rule out CVT [6].

In our study, the cerebrospinal fluid (CSF) mostly showed abnormal composition and elevation of the intracranial pressure. The CSF assessment revealed associated purulent meningitis in two patients, meningitis with an increased number of lymphocytes and red cells, an isolated elevation of red cells at two patients. However, we did not find any elevation of proteins in CSF. The opening pressure was elevated in all patients except the cases of cavernous sinus thrombosis. It varies between 19 and 48 cm H2O.

Detailed CT scan findings have been described in earlier literature [7–10]. The CT scan without and with of contrast agent injection remains the first exam achieved whenever a CVT is suspected. It provides the diagnosis proof and allows showing the direct signs of CVT. However, up to 20% CVT cases have shown a normal CT [11], more frequently they are patients with isolated intracranial hypertension. Therefore, a CVT diagnosis has to be systematically discarded before retaining an idiopathic intracranial hypertension diagnosis [12, 13]. However, the CT scan sensitivity was 30% in Ameri study [14]. The empty delta sign (the frequent direct sign) was found in six patients (23%) compared to 20% found in the literature [15]. The empty lateral sinus sign is the equivalent of the empty delta sign. This seemed to be underestimated in the literature [16], whereas it represents the first direct sign in our study and was found in 11 patients (37%). It is necessary to differentiate it from hypoplasic sinus mainly the left lateral sinus. A special attention must be allocated to the signs of the transverse sinus on the mastoid in images before contrast agent injection.

Indirect signs are more frequent and less specific compared to direct signs in different studies. This was also confirmed in our study (85%). Most often, the CT-scan shows thrombosis consequences on the cerebral parenchyma under the shape of a hypodensity (oedema or venous infarction), or a hyperdensity bound to a haemorrhage going from some small patches to a real intraparenchymal haematoma. The infarct is frequently hemorrhagic (10 to 50% of cases in the literature) [17]. In our study, the CT scan was sufficient to diagnose 50% of cases at the admission.

Currently, the gold standard in the disagnosis of CVT is to combine MRI and MRV for visualizing the thrombosed vessel. The indirect signs of CVT shown in the MRI are similar to those in the CT scan since they are not specific; both might withdraw 25% of cases. The sensitivity of the MRI associating the MRV is 90% in Lafitte study [18]. The MRI and the MRV allowed the diagnosis in all cases of our series (56%). The MRI alone is limited by flow artifacts that can lead to false positives and the absence of hyperintense signal on T1 and T2-weighted images at the onset of acute thrombosis [19]. During the first 3 to 5 days the thrombosed sinus is isointense on T1 and hypointense on T2, and thus very difficult to differentiate from normal veins. All MRI examinations have been achieved after the 5th day of the onset. For all cases; (except those with deep veins thrombosis, cortical veins thrombosis and the cavernous sinus thrombosis); the thrombosed sinus was hyperintense on T1 and hyper or isointense on T2. MRV done alone cannot discriminate hypoplasia than lateral sinus thrombosis [20]. It is illustrated by one case (case number 5) that shows an amputation of the hypoplasic LLS confirmed by morphological T2 and T1 sequences. The cortical or deep vein thrombosis might be misdiagnosed even by MRI and MRV. This fact occurred in one case presented with a deep vein thrombosis (case number 18).

The diagnosis of isolated cortical vein thrombosis might be difficult, requiring conventional angiography. In our series, conventional angiography investigation was not used. The Echo-planar susceptibility-weighted images (T2*) are particularly useful in isolated cortical venous thrombosis and during early stage of acute CVT when T1 and T2 lack sensitivity [21]. the diagnosis of cortical venous thrombosis was performed by echo-planar susceptibility-weighted images (T2*) at one patient (case number 20) (Figure 4).

The thrombosis of the cavernous sinus was revealed in three cases by an absence of heightening of one or the two cavernous sinuses in the coronal T1 images after Gadolinium injection, it was the best to show the sinus thrombosis. Two patients had cavernous sinus thrombosis associated with intracavernous carotid aneurysm.

CVT is often multifactorial since several disorders might initiate or dispose patients. In the International studies of the Cerebral Vein and Dural Sinus Thrombosis (ISCVT), 44% of the patients had shown more than one origin and predisposing factor including the congenital and genetic thrombophilia which was recorded in 22% patients [22]. In developped countries, non-infectious origins such oral contraceptives, cancer, and thrombophilia are the most frequent causes with a prevalence of 80% [4, 23]. The etiologies in our study was similar to the developing countries findings [24, 25]. Infections and post-partum causes are the most frequent causes. Local infectious etiologies are observed in seven cases including four cases of otomastoiditis, three cases of severe staphylococcal of the face and one case of ethmoiditis. CVT in these cases may be explained by the consultation delay of our patients. CVT represents 23.5% of the stroke during pregnancy and postpartum [26]. Several factors could be associated to the postpartum CVT, these included caesarean section, arterial hypertension, and anemia. On the other hand, eclampsia is not a risk factor. Indeed no case of eclampsia has been counted in our study [27]. In one case, CVT occurred after epidural analgesia. Oral contraceptives represent 12% in Biousse and Bousser study. In our context, we are convinced that some cases are not diagnosed considering the increasing use of the oral contraceptives in our environment. Hemostasis investigation includes congenital or genetic thrombophilia (deficiencies in antithrombin III, protein C, and protein S and having the factor V Leiden or prothrombin gene mutations, and antiphospholipid antibodies). We have investigated thrombophilia in four cases; one case of deficiency in protein C, and two cases in protein S were revealed. The protein S deficiency was not confirmed by a second dosage. Congenital thrombophilia is certainly underestimated in our study. CVT due to Behcet disease is frequent in Mediterranean countries, but Behcet's cases seem less frequent in our study compared with Daif study. The low frequency of Behçet disease might be explained by our limited cases. CVT was idiopathic in 20% in agreement with literature data [28].

Heparin (low-molecular-weight heparin or standard heparin) is the first-line treatment even in presence of hemorrhagic infarction [29, 30]. In our study, 90% of patients benefited from anticoagulant treatment including those with hemorrhagic infarction. Systemic or local thrombolysis may be used in CVT when patients deteriorate despite adequate anticoagulation and other causes of deterioration have been ruled out, thrombolysis or thrombectomy may be considered in specialized centers [31, 32]. In patients with isolated intracranial hypertension, a lumbar puncture for removing the cerebrospinal fluid is required before starting heparin. If intracranial pressure is severely raised, the general recommendations should be followed, this starts with mannitol treatment to admission to an Intensive Care Unit with intracranial pressure monitoring or even decompressive hemicraniectomy when a risk of cerebral herniation exists. In our patients who consulted at a stage of important decrease of visual cuit,y we used carbonic anhydrase inhibitor or mannitol or even steroids although they are not recommended by most authors [33]. Antiepileptics and antibiotics are useful to treat seizures and infection. One of our patient underwent a partial epilepsy which was stabilized under carbamazepin. The advisable anticoagulant treatment duration is 6 to 12 months. For our patients, the mean duration of anticoagulation therapy was 6 months. ISCVT shows that death and severe sequelaes were observed in 15% of patients [22]. In our study, the result was similar to ISCVT (13% vs 15%)with two cases of severe sequelaes and two cases of death.

## Conclusion

CVT is not uncommon in Morocco. Post-partum and infectious causes are more frequent in this study. Behcet's disease etiology seems to be underestimated. The investigation of the haemostasis should be a routine after each cerebral venous thrombosis. MRI and Venous MR angiography are the key diagnostic tools. CT scan should be more effective for establishing the CVT diagnosis. The outcome is favorable using heparin treatment.

## References

[1] Marie-Germaine Bousser, José M Ferro (2007). Cerebral venous thrombosis: an update. Lancet Neurol..

[2] Cumurciuc R, Crassard I, Sarov M, Valade D, Bousser MG (2005). Headache as the only neurological sign of cerebral venous thrombosis: a series of 17 cases. J Neurol Neurosurg Psychiatry..

[3] Diener HC (2005). J. Neurol. Cerebral venous thrombosis? headache is enough. Neurosurg. Psychiatry.

[4] Einhäupl KM, Villringer A, Habert RL, Einhäupl KM, Kempski O, Baethmann A (1990). Clinical spectrum of sinus venous thrombosis. Cerebral sinus thrombosis: experimental and clinical aspects.

[5] Lamy C (2008). Epilepsie et accident vasculaire cerebral. Revue neurologique..

[6] Crassard Isabelle, Soria Claudine, Tzourio Christophe, Woimant France (2005). A Negative D-Dimer Assay Does Not Rule Out Cerebral Venous Thrombosis. A Series of Seventy-Three Patients. Stroke.

[7] Chiras J, Bousser MG, Meder JF, Kouss A, Bories JC Tin (1985). Cerebral thrombophlebitis. Neuroradiology..

[8] Goldberg AL, Rosenbaum AE, Wang H, Kim WS, Lewis VL, Hanley DF (1986). Computed tomography of dural sinus thrombosis. J Comput Assist Tomogr..

[9] Perkin GD (1995). Cerebral venous thrombosis: developments in imaging and treatment. J Neurol Neurosurg Psychiatry..

[10] Casey SO, Alberico RA, Patel M (1996). Cerebral CT venography. Radiology..

[11] Bousser MG, Russell RR (1997). Cerebral venous thrombosis.

[12] Khandalwal S, Miller CD (2004). Distinguishing dural sinus thrombosis from benign intracranial hypertension. Emerg Med J..

[13] Biousse V, Ameri A, Bousser MG (2000). Isolated intracranial hypertension as the only sign of cerebral venous thrombosis. Neurology..

[14] Ameri A, Bousser MG (1992). Cerebral venous thrombosis. Neurol Clin..

[15] Virapongse C, Cazenave C, Quisling R, Sarwar M, Hunter S (1987). The empty delta sign: frequency and significance in 76 cases of dural sinus thrombosis. Radiology..

[16] Arquizan C (2001). Thrombophlébites cérébrales: aspects cliniques, diagnostic et traitement. Réanimation..

[17] Crassard I, Ameri A, Rougemont D, Bousser MG (2003). Thromboses veineuses cérébrales. Encycl Méd Chir Neurologie..

[18] Lafitte F (1997). MRI and MRA for Diagnosis and Follow-up of cerebral Venous Thrombosis. Clinical Radiology..

[19] Bousser MG (2000). Cerebral venous thrombosis: diagnosis and management. J Neurol..

[20] Ayanzen RH (2000). Cerebral MR Venography: Normal Anatomy and Potential Diagnostic Pitfalls. AJNR Am J Neuroradiol..

[21] Idbaih A, Boukobza M, Crassard I, Porcher R, Bousser MG, Chabriat H (2006). MRI of clot in cerebral venous thrombosis high diagnostic value of susceptibility-weighted images. Stroke.

[22] Ferro José M, Canhão Patrícia, Stam Jan, Bousser Marie-Germaine, Barinagarrementeria Fernando (2004). Prognosis of Cerebral Vein and Dural Sinus Thrombosis Results of the International Study on Cerebral Vein and Dural Sinus Thrombosis (ISCVT). Stroke..

[23] Biousse V, Bousser MG (1999). Cerebral venous thrombosis. Neurologist..

[24] Zhang Zaiqiang (2000). Cerebral venous sinus thrombosis: a clinical study of 23 cases. Chinese Medical journal..

[25] Daif Abdulkader (1995). Cerebral Venous Thrombosis in Adults: A Study of 40 Cases From Saudi Arabia. Stroke..

[26] Cantu Carlos, Barinagarrementeria Fernando (1993). Cerebral Venous Thrombosis Associated With Pregnancy and Puerperium Review of 67 Cases. Stroke..

[27] Douglas J Lanska, Richard J Kryscio (2000). Risk Factors for Peripartum and Postpartum Stroke and Intracranial Venous Thrombosis. Stroke..

[28] Crassard I, Bousser M.G (2005). Céphalées au cours des thromboses veineuses cérébrales. Rev Neurol (Paris).

[29] De Bruijn SFTM, Stam J (1999). Randomized, Placebo-Controlled Trial of Anticoagulant Treatment With Low-Molecular-Weight Heparin for Cerebral Sinus Thrombosis. Stroke..

[30] Pillai Lalitha V, Ambike Dhananjay P (2005). Cerebra venous thrombosis: an experience with anticoagulation with low molecular weight heparin. Indian journal of critical care medicine..

[31] Ciccone Alfonso, Canha O Patricia, Falca O Filipa, Ferro José M, Sterzi Roberto (2004). Thrombolysis for Cerebral Vein and Dural Sinus Thrombosis. Stroke..

[32] Wasay M, Bakshi R, Kojan S, Bobustuc G, Dubey N, Unwin DH (2001). Nonrandomized Comparison of Local Urokinase Thrombolysis Versus Systemic Heparin Anticoagulation for Superior Sagittal Sinus Thrombosis. Stroke..

[33] Canhão P, Cortesão A, Cabral M (2004). Are steroids useful for the treatment of cerebral venous thrombosis: ISCVT results. Cerebrovasc Dis..

